# Supplementary material for: EpiAlign: an alignment-based bioinformatic tool for comparing chromatin state sequences
Source: Nucleic Acids Res. 2019 Apr 24;47(13):e77. doi: 10.1093/nar/gkz287 (PMC6648345; doi:10.1093/nar/gkz287)
Supplement: gkz287_Supplemental_File [file gkz287_supplemental_file.pdf]

Supplementary Materials of "EpiAlign: an alignment-based bioinformatic tool for comparing chromatin state sequences"

1 Frequency-based weights

The frequency-based weight of the k-th state in chromatin state sequence S is $W^{Fb,w}(k,S)$, which is defined as:

$$W^{Fb,w}(k,S) = GS^{w}(k,S) \cdot RS^{w}(k,S) \cdot LS(k,S)$$

where w is a window size parameter, $GS^{w}(k,S)$ is the two-state frequency score, $RS^{w}(k,S)$ is the one-state frequency score, and $LS(k,S)$ is the length score.

In the subsequence $S^{[k-w,k+w]}$, if neither of pattern $S^{[k-1,k]}$ or $S^{[k,k+1]}$ occur elsewhere in the subsequence, $GS^{w}(k,S) = 1$; if both patterns occur somewhere in the subsequence, $GS^{w}(k,S) = 4$; otherwise $GS^{w}(k,S) = 2$.

The one-state frequency score $RS^{w}(k,S)$ represents the frequency of each state and gives more frequent states smaller scores. For $S^{[k-w,k+w]}$, we rank the chromatin states in this window by their frequencies, from the highest to the lowest. Then

$$RS^{w}(k,S) = 1 + \frac{\text{rank}(S^{[k]}) - 1}{\text{number of unique states in } S^{[k-w,k+w]} - 1} \in [1,2] \,.$$

In the compression process of EpiAlign, we compress consecutive occurrences of the same state into a state label. We also obtain an occurrence number for each state. For example, a chromatin state sequence `abbcc` is represented by a compressed state sequence $S =$ `abc` and a state occurrence sequence $L =$ `122`. The length score $LS(k,S)$ is based on the occurance sequence L. If $L^{[k]} = 1$, $LS(k,S) = 0.5$; otherwise $LS(k,S) = 1$.

2 Average length of stretches of the same chromatin state

The motivation for doing compression before alignment comes from the fact that most uncompressed sequences contain long stretches of the same chromatin state. Here we use letter "a" to "o" to denote chromatin state 1 to 15 from the Roadmap 15-state annotation. We calculate the average length of consecutive same chromatin state for each state. From the results in Figure S1, we can see that the chromatin state "o", which means quiescent/low state, is much longer than other states before compression. As the length information of such a state is hardly biologically meaningful. The compression step is needed for addressing this issue by turning the focus onto the more biologically meaningful chromatin state patterns.

3 Horizontal alignment scores

It is worth noting that the alignment scores of multiple query chromatin state sequences are not directly comparable. To normalize the alignment scores, we align every query chromatin state

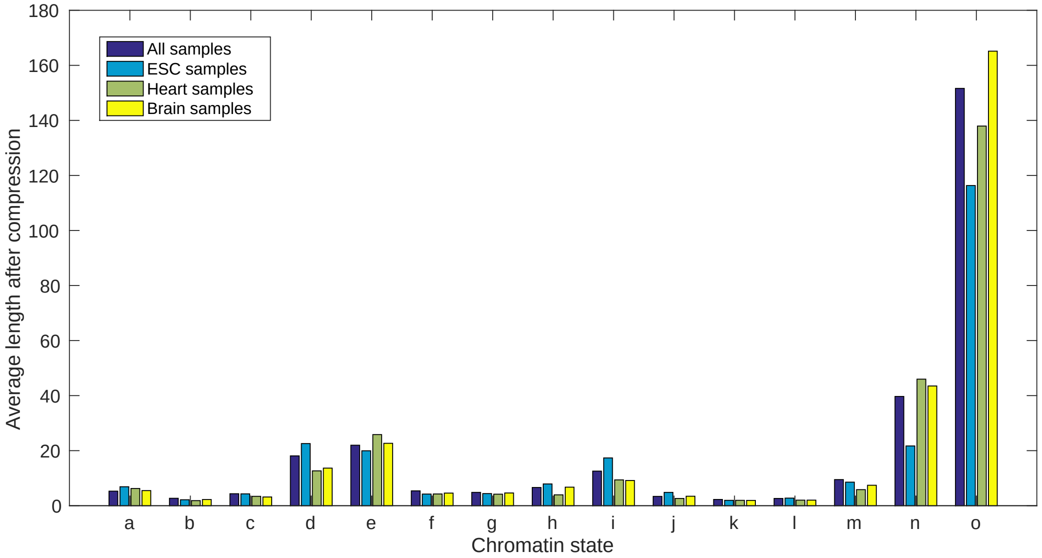

Figure S1: Average lengths of stretches of a single chromatin state in ESC, heart and brain samples. Letters 'a' to 'o' refer to chromatin states 1 to 15 in the Roadmap 15-state chromHMM annotation. For each state, we calculated the average length of its stretches, i.e., consecutive occurrences. It is obvious that the chromatin state 'o', i.e., the quiescent/low state, has much longer stretches than other states do.

sequence to randomized chromatin state sequences, which serve as a negative control. For each region in the real epigenome, this region is used as the "query" and aligned to each of the three randomized epigenomes to obtain its hit in that randomized epigenome. Here the randomized epigenomes have the same lengths as their real counterparts and are generated by the Markov rule, with a state transition probability matrix per chromosome based on the real epigenome. The alignment scores of the three hits are then averaged as the baseline score of this query region. We use Q_i to denote the alignment score of region i's hit in the real epigenome, P_i to denote the baseline score of region i, and define the horizontal alignment score of region i as $\frac{Q_i - Pi}{Pi}$. A high score indicates that the region shares a highly similar and non-random chromatin state sequence with another region in the same epigenome, implying that the region's chromatin state sequence pattern is likely biologically meaningful.

4 Generation of hybrid epigenomes

For every chromosome, we first divide both its real chromatin state sequence ("epigenome") and their randomized counterparts into non-overlapping regions of 50 million bp length. Then for the i-th region in the hybrid epigenome, its chromatin state sequence is set as the sequence of the i-th region in the real epigenome when i is even, or as the sequence of the i-th region in the randomized epigenome when i is odd. We can easily generate another hybrid epigenome if we switch the odd and even regions.

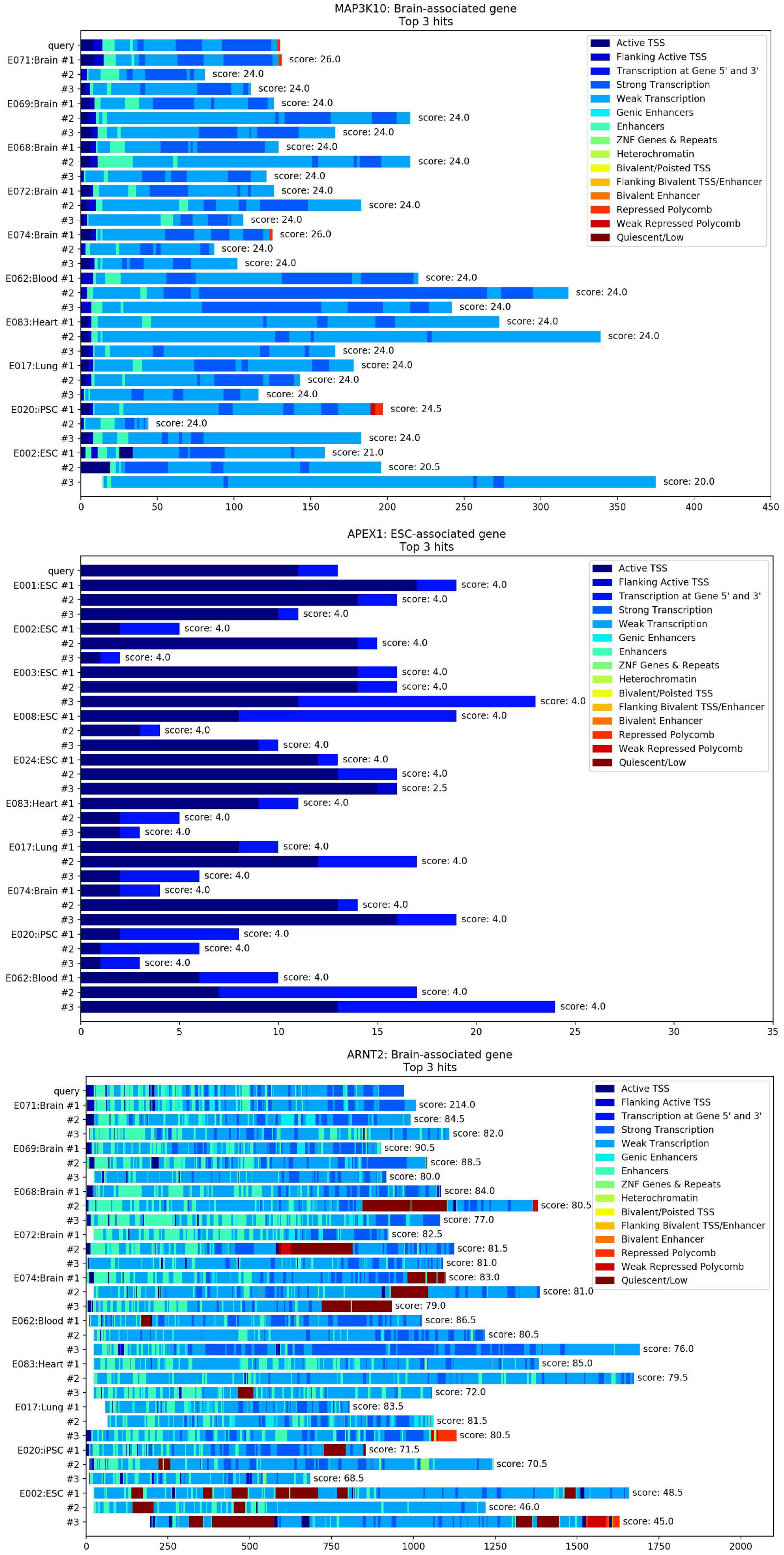

Figure S2: Top hits of 3 different queries in epigenomes from various tissues. The queries are chromatin state sequences of a brain-associated gene *MAP3K10*, an ESC-associated gene *APEX1*, and a brain-associated gene *ARNT2*.

5 Visualization of top hits in horizontal alignment

Figure S2 shows that when we use the chromatin state sequences of gene regions as queries, the best hits (regions that have the highest horizontal alignment scores with the query) reported by EpiAlign are very similar to the query in terms of chromatin state patterns.

6 Comparison of uncompressed sequences and compressed sequences in Vertical Alignment

We also use the vertical alignment to justify our choice of aligning compressed chromatin state sequences instead of original uncompressed sequences. We repeated the vertical alignment analysis on all brain-specific genes and all heart-specific genes among the brain and heart samples, using the uncompressed sequences instead of the compressed chromatin state sequences. Then we performed the same two-sample one-sided Wilcoxon test between brain-vs-brain alignment scores and brain-vs-heart alignment scores on these selected genes, and we denote the resulting p-values as uncompressed p-values. Next we compare these uncompressed p-values with their corresponding p-values we obtain previously based on the compressed sequences. We counted the number of significantly different genes, which have a p-value less than 0.05 after Bonferroni correction, from both analyses. From compressed sequences, 112 out of 327 tissue-specific genes are significant and from uncompressed 120 out of 327 are significant. We also conducted two-sample Wilcoxon test between the original p-values and p-values from uncompressed sequences. The result shows no significant difference in distribution (p-value = 0.509). These results show that the resulting p-values from the compressed sequences are very similar to those from the uncompressed sequences. Considering that alignment of uncompressed sequences is much more time-consuming (takes 20 times more time than compressed sequences), the compression step makes the alignment algorithm more effective.

7 Examples of vertical alignment on tissue-associated genes

Since epigenetic marks carry important regulatory information relevant to cell differentiation, chromatin states learned from these marks should also contain cell-type characteristic patterns. For a tissue-associated gene, we should expect to observe significantly higher similarity of chromatin states within its associated cell type than the counterpart similarity between cells of other cell types. We implement vertical alignment on the Roadmap dataset on some tissue-associated genes [1]. Taken an ESC-associated gene *ANAPC1* as an example, we use the alignment scores calculated by EpiAlign to compare the similarity of *ANAPC1*'s chromatin state sequences in different cell types. We first extract the chromatin state sequence of *ANAPC1*'s chromatin region from in each epigenome. Then, we use EpiAlign to calculate the alignment scores of these chromatin state sequences between each pair of the 127 epigenomes, resulting in $8,001$ pairwise alignment scores in total. We consider these $8,001$ alignment scores as the population and refer to the alignments scores between epigenomes of the same cell type (i.e., ESC) as the Group A, and the whole population as the Group B. As there are 8 ESC epigenomes, we obtain 28 alignment scores in the Group A and then calculate the percentile of each of these 28 scores in the population. As shown in Figure S3, 11 out of the 28 scores are among the upper 5% percentile, and the average percentile of alignment scores in group A is 0.256. We also perform a one-tailed t-test to compare the alignment scores of the two groups. The p-value of the test is 0.001, which suggests that the chromatin states corresponding to the ESC-associated gene *ANAPC1* have more similar patterns among ESC samples

in comparison with the other cell types in the Roadmap dataset. We also use the alignment scores based on the naïve method and repeat all the analysis above.

From Figure S3, we can see that compared to the naïve method, EpiAlign can better distinguish alignment scores among ESCs from others. Similar results are observed for brain and heart too. These results indicate that for a specific cell type, EpiAlign is able to detect the similarity of chromatin states of its tissue-associated genes, suggesting that EpiAlign may be used to differentiate a given cell type from the other tissue and cell types, by evaluating the similarity of epigenetic signals on its associated genes. Also, these results show that EpiAlign can be used to identify tissue-associated regions.

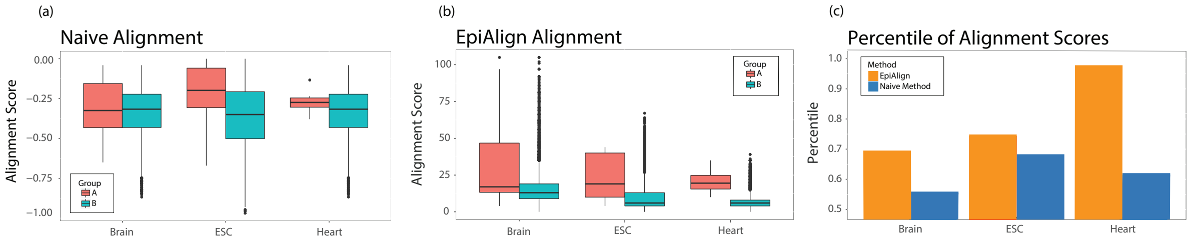

Figure S3: (a)-(b) Boxplots of pairwise alignment scores of chromatin state sequences of a tissue-associated gene within same the cell type (Group A) and across all samples (Group B). We choose an ESC-associated gene *ANAPC1*, a brain-associated gene *AK5*, and a heart-associated gene *ACTN2*. (a) shows the alignment scores by the naïve method, and (b) shows the EpiAlign alignment scores. (c) shows the average percentile of group A scores in Group B for each alignment method.

We also perform hierarchical clustering of the 127 cells using chromatin state sequences of multiple tissue-associated genes. For example, for each of the 118 ESC-associated genes, we use EpiAlign to calculate all the pairwise alignment scores and form a 127×127 score matrix. We then normalize the scores by dividing the maximum so that for each gene i, we get a 127×127 normalized comparison matrix M^i. Then the final distance matrix D is calculated as $D_{jk} = -\sqrt{\sum_{i=1}^{118} M_{jk}^i}$. Finally, we perform complete-linkage hierarchical clustering on the 127 epigenomes based on the distance matrix. The heatmap of the distance matrix and the clustering results are shown in Figure S4(b). The heatmap can roughly distinguish ESC samples from the other cell types. In hierarchical clustering, 7 out of 8 ESC samples are successfully grouped together when the cluster number is set as 10. Similarly, we perform the above analysis based on brain-associated genes, and the heatmap is shown in S4(a). The brain samples can be clearly differentiated. In addition, all the 10 brain samples are successfully grouped together by hierarchical clustering when cluster number is set as 10. The above results confirm EpiAlign's capability to search for similar chromatin state patterns and suggest that chromatin states of the same genomic region are more similar within cell types.

8 Male-vs-female vertical alignment

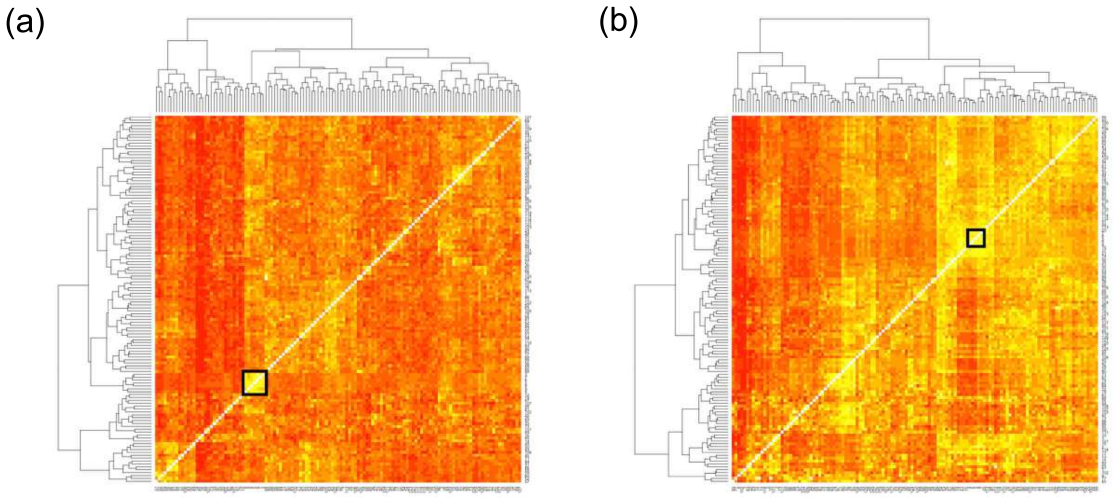

Figure S4: Clustering results using (a) brain-associated genes or (b) ESC-associated genes. Samples in black boxes are (a) brain samples and (b) ESC samples.

9 Brain-vs-heart vertical alignment

9.1 Genes with the smallest p-values from one-sided Wilcoxon test

We perform the two-sample one-sided Wilcoxon test between the brain-vs-brain alignment scores and the brain-vs-heart alignment scores for all the protein-coding genes. The top 200 genes that we use to perform the gene ontology enrichment analysis are listed in Table S2.

	Gene.stable.ID	Chromosome	Gene.name	Strand	Gene.start..bp.	Gene.end..bp.
1	ENSG00000004700	12	RECQL	-1	21468911	21501669
2	ENSG00000006047	17	YBX2	-1	7288252	7294615
3	ENSG00000015592	8	STMN4	-1	27235323	27258420
4	ENSG00000033122	1	LRRC7	1	69568398	70151945
5	ENSG00000034053	15	APBA2	1	28884483	29118315
6	ENSG00000047365	4	ARAP2	-1	35948221	36244509
7	ENSG00000050438	12	SLC4A8	1	51391317	51515763
8	ENSG00000054282	1	SDCCAG8	1	243256034	243500092
9	ENSG00000056487	22	PHF21B	-1	44881162	45009999
10	ENSG00000064270	16	ATP2C2	1	84368527	84464187
11	ENSG00000065609	6	SNAP91	-1	83552880	83709691
12	ENSG00000066468	10	FGFR2	-1	121478334	121598458
13	ENSG00000067221	15	STOML1	-1	73978923	73994622
14	ENSG00000067798	12	NAV3	1	77324641	78213008
15	ENSG00000070501	8	POLB	1	42338454	42371808
16	ENSG00000073803	3	MAP3K13	1	185282941	185489097
17	ENSG00000074211	4	PPP2R2C	-1	6320578	6563600
18	ENSG00000077009	19	NMRK2	1	3933103	3942416
19	ENSG00000078295	5	ADCY2	1	7396208	7830081
20	ENSG00000078725	9	BRINP1	-1	119153458	119369467
21	ENSG00000084628	1	NKAIN1	-1	31179745	31239554

22	ENSG00000088766	20	CRLS1	1	6006090	6040053
23	ENSG00000089225	12	TBX5	-1	114353931	114408442
24	ENSG00000091129	7	NRCAM	-1	108147623	108456717
25	ENSG00000095397	9	WHRN	-1	114402080	114505450
26	ENSG00000100290	22	BIK	1	43110748	43129712
27	ENSG00000100433	14	KCNK10	-1	88180103	88326907
28	ENSG00000100505	14	TRIM9	-1	50975262	51096061
29	ENSG00000102383	X	ZDHHC15	-1	75368427	75523502
30	ENSG00000104112	15	SCG3	1	51681353	51721031
31	ENSG00000104833	19	TUBB4A	-1	6494319	6502848
32	ENSG00000105048	19	TNNT1	-1	55132794	55149354
33	ENSG00000106780	9	MEGF9	-1	120600813	120714470
34	ENSG00000107438	10	PDLIM1	-1	95237572	95291024
35	ENSG00000108001	10	EBF3	-1	129835283	129963841
36	ENSG00000108187	10	PBLD	-1	68282660	68333049
37	ENSG00000108688	17	CCL7	1	34270221	34272242
38	ENSG00000108830	17	RND2	1	43025241	43032036
39	ENSG00000109472	4	CPE	1	165361194	165498320
40	ENSG00000109654	4	TRIM2	1	153152342	153339320
41	ENSG00000109956	11	B3GAT1	-1	134378504	134411918
42	ENSG00000110042	11	DTX4	1	59171430	59208587
43	ENSG00000110076	11	NRXN2	-1	64606174	64723188
44	ENSG00000110628	11	SLC22A18	1	2899721	2925246
45	ENSG00000111605	12	CPSF6	1	69239537	69274358
46	ENSG00000111726	12	CMAS	1	22046174	22065674
47	ENSG00000112041	6	TULP1	-1	35497874	35512938
48	ENSG00000112139	6	MDGA1	-1	37630679	37699306
49	ENSG00000112290	6	WASF1	-1	110099819	110180004
50	ENSG00000112379	6	ARFGEF3	1	138161921	138344663
51	ENSG00000113456	5	RAD1	-1	34905264	34918989
52	ENSG00000113460	5	BRIX1	1	34915376	34925996
53	ENSG00000113645	5	WWC1	1	168291651	168472303
54	ENSG00000115041	2	KCNIP3	1	95297304	95386083
55	ENSG00000115239	2	ASB3	-1	53532672	53787610
56	ENSG00000117020	1	AKT3	-1	243488233	243851079
57	ENSG00000117595	1	IRF6	-1	209785623	209806175
58	ENSG00000118322	5	ATP10B	-1	160563120	160852214
59	ENSG00000120937	1	NPPB	-1	11857464	11858931
60	ENSG00000120963	8	ZNF706	-1	101177878	101206193
61	ENSG00000121058	17	COIL	-1	56938187	56961054
62	ENSG00000121743	13	GJA3	-1	20138255	20161049
63	ENSG00000121904	1	CSMD2	-1	33513999	34165842
64	ENSG00000123560	X	PLP1	1	103773718	103792619
65	ENSG00000124641	6	MED20	-1	41905354	41921139
66	ENSG00000127955	7	GNAI1	1	79768028	80226181
67	ENSG00000128524	7	ATP6V1F	1	128862826	128865844
68	ENSG00000129250	17	KIF1C	1	4997948	5028401
69	ENSG00000129991	19	TNNI3	-1	55151767	55157773

70	ENSG00000130176	19	CNN1	1	11538717	11550323
71	ENSG00000130226	7	DPP6	1	153887097	154894285
72	ENSG00000130475	19	FCHO1	1	17747718	17788568
73	ENSG00000131409	19	LRRC4B	-1	50516892	50568045
74	ENSG00000131437	5	KIF3A	-1	132692628	132737638
75	ENSG00000132549	8	VPS13B	1	99013266	99877580
76	ENSG00000133216	1	EPHB2	1	22710839	22921500
77	ENSG00000133958	14	UNC79	1	93333219	93707876
78	ENSG00000135069	9	PSAT1	1	78297143	78330093
79	ENSG00000135269	7	TES	1	116210493	116258783
80	ENSG00000135298	6	ADGRB3	1	68635367	69389511
81	ENSG00000136155	13	SCEL	1	77535674	77645263
82	ENSG00000136193	7	SCRN1	-1	29920103	29990289
83	ENSG00000136574	8	GATA4	1	11676959	11760002
84	ENSG00000137266	6	SLC22A23	-1	3268962	3457022
85	ENSG00000139364	12	TMEM132B	1	125186836	125662377
86	ENSG00000140937	16	CDH11	-1	64943753	65126112
87	ENSG00000141448	18	GATA6	1	22169443	22202528
88	ENSG00000141574	17	SECTM1	-1	82321024	82334074
89	ENSG00000141738	17	GRB7	1	39737927	39747291
90	ENSG00000142949	1	PTPRF	1	43525187	43623666
91	ENSG00000143951	2	WDPCP	-1	63121383	63827843
92	ENSG00000144369	2	FAM171B	1	186693971	186765965
93	ENSG00000144857	3	BOC	1	113211003	113287459
94	ENSG00000145284	4	SCD5	-1	82629539	82798857
95	ENSG00000145555	5	MYO10	-1	16661914	16936276
96	ENSG00000145794	5	MEGF10	1	127290831	127465737
97	ENSG00000146005	5	PSD2	1	139795821	139844466
98	ENSG00000146352	6	CLVS2	1	122995971	123072927
99	ENSG00000147488	8	ST18	-1	52110839	52460959
100	ENSG00000147724	8	FAM135B	-1	138130023	138496822
101	ENSG00000147799	8	ARHGAP39	-1	144529179	144605816
102	ENSG00000148123	9	PLPPR1	1	101028709	101325135
103	ENSG00000149571	11	KIRREL3	-1	126423359	127003460
104	ENSG00000149596	20	JPH2	-1	44111695	44187578
105	ENSG00000150477	18	KIAA1328	1	36829106	37232172
106	ENSG00000150625	4	GPM6A	-1	175632934	176002664
107	ENSG00000152578	11	GRIA4	1	105609994	105982092
108	ENSG00000154229	17	PRKCA	1	66302636	66810743
109	ENSG00000155886	9	SLC24A2	-1	19507452	19786928
110	ENSG00000156475	5	PPP2R2B	-1	146581146	147084784
111	ENSG00000157103	3	SLC6A1	1	10992186	11039249
112	ENSG00000157423	16	HYDIN	-1	70807378	71230722
113	ENSG00000157851	2	DPYSL5	1	26847747	26950351
114	ENSG00000158014	1	SLC30A2	-1	26037252	26046133
115	ENSG00000158615	1	PPP1R15B	-1	204403387	204411791
116	ENSG00000162706	1	CADM3	1	159171609	159203313
117	ENSG00000163449	2	TMEM169	1	216081866	216102783

118	ENSG00000164107	4	HAND2	-1	173524969	173530229
119	ENSG00000164163	4	ABCE1	1	145097932	145129179
120	ENSG00000164532	7	TBX20	-1	35202430	35254147
121	ENSG00000164542	7	KIAA0895	-1	36324221	36390125
122	ENSG00000165312	10	OTUD1	1	23439458	23442390
123	ENSG00000165527	14	ARF6	1	49893092	49897054
124	ENSG00000165548	14	TMEM63C	1	77116568	77259495
125	ENSG00000165566	13	AMER2	-1	25161684	25172288
126	ENSG00000166501	16	PRKCB	1	23835946	24220611
127	ENSG00000166831	15	RBPMS2	-1	64739892	64775587
128	ENSG00000166922	15	SCG5	1	32641676	32697098
129	ENSG00000167553	12	TUBA1C	1	49188736	49274603
130	ENSG00000168280	2	KIF5C	1	148875250	149026759
131	ENSG00000168495	8	POLR3D	1	22245104	22254600
132	ENSG00000168958	2	MFF	1	227325151	227357836
133	ENSG00000170091	5	NSG2	1	174045604	174243501
134	ENSG00000170185	4	USP38	1	143184917	143223830
135	ENSG00000171954	19	CYP4F22	1	15508493	15552317
136	ENSG00000172379	15	ARNT2	1	80404350	80597937
137	ENSG00000172461	6	FUT9	1	96015984	96215612
138	ENSG00000172995	3	ARPP21	1	35638945	35794496
139	ENSG00000173530	8	TNFRSF10D	-1	23135588	23164030
140	ENSG00000173898	11	SPTBN2	-1	66685248	66729226
141	ENSG00000174099	12	MSRB3	1	65278643	65491430
142	ENSG00000174407	20	MIR1-1HG	1	62550453	62570764
143	ENSG00000174672	11	BRSK2	1	1389899	1462689
144	ENSG00000175084	2	DES	1	219418377	219426739
145	ENSG00000175087	1	PDIK1L	1	26111165	26125543
146	ENSG00000175161	3	CADM2	1	84958981	86074429
147	ENSG00000176049	5	JAKMIP2	-1	147585439	147782848
148	ENSG00000177103	11	DSCAML1	-1	117427773	117817525
149	ENSG00000177508	16	IRX3	-1	54283304	54286763
150	ENSG00000177807	1	KCNJ10	-1	159998651	160070483
151	ENSG00000178445	9	GLDC	-1	6532464	6645783
152	ENSG00000179242	20	CDH4	1	61252426	61940617
153	ENSG00000179314	17	WSCD1	1	6057807	6124427
154	ENSG00000179915	2	NRXN1	-1	49918505	51225575
155	ENSG00000180287	1	PLD5	-1	242082986	242524696
156	ENSG00000182600	2	SNORC	1	232857270	232878708
157	ENSG00000183072	5	NKX2-5	-1	173232109	173235357
158	ENSG00000185155	1	MIXL1	1	226223618	226227054
159	ENSG00000185156	17	MFSD6L	-1	8797162	8799349
160	ENSG00000185565	3	LSAMP	-1	115802363	117139389
161	ENSG00000185627	11	PSMD13	1	236546	252984
162	ENSG00000185818	4	NAT8L	1	2059512	2069089
163	ENSG00000185973	X	TMLHE	-1	155490115	155669944
164	ENSG00000186231	6	KLHL32	1	96924620	97140754
165	ENSG00000187164	10	SHTN1	-1	116881482	117126586

166	ENSG00000187634	1	SAMD11	1	923928	944581
167	ENSG00000188015	1	S100A3	-1	153547329	153549372
168	ENSG00000188316	10	ENO4	1	116849512	116911788
169	ENSG00000188522	17	FAM83G	-1	18968789	19004804
170	ENSG00000196220	3	SRGAP3	-1	8980591	9363053
171	ENSG00000196338	X	NLGN3	1	71144831	71171201
172	ENSG00000196361	19	ELAVL3	-1	11451326	11481046
173	ENSG00000196376	6	SLC35F1	1	117907526	118317676
174	ENSG00000196581	1	AJAP1	1	4654732	4792534
175	ENSG00000196628	18	TCF4	-1	55222331	55664787
176	ENSG00000196767	X	POU3F4	1	83508261	83512127
177	ENSG00000197728	12	RPS26	1	56041351	56044697
178	ENSG00000198216	1	CACNA1E	1	181317690	181808084
179	ENSG00000198513	14	ATL1	1	50532509	50633068
180	ENSG00000198732	14	SMOC1	1	69854131	70032366
181	ENSG00000203930	X	LINC00632	1	140709562	140793215
182	ENSG00000204011	9	COL5A1-AS1	-1	134649385	134652843
183	ENSG00000204344	6	STK19	1	31971091	31982821
184	ENSG00000204624	1	DISP3	1	11479166	11537584
185	ENSG00000204683	10	C10orf113	-1	21125763	21146559
186	ENSG00000205758	21	CRYZL1	-1	33589341	33643926
187	ENSG00000213578	15	CPLX3	1	74826547	74831802
188	ENSG00000214160	3	ALG3	-1	184242301	184249548
189	ENSG00000214338	6	SOGA3	-1	127472794	127519191
190	ENSG00000214595	2	EML6	1	54723499	54972025
191	ENSG00000219438	22	FAM19A5	1	48489460	48850912
192	ENSG00000221818	8	EBF2	-1	25841730	26045397
193	ENSG00000235568	22	NFAM1	-1	42380410	42432395
194	ENSG00000237330	1	RNF223	-1	1070966	1074307
195	ENSG00000241370	6	RPP21	1	30345131	30346884
196	ENSG00000243232	5	PCDHAC2	1	140966235	141012344
197	ENSG00000243449	4	C4orf48	1	2041993	2043970
198	ENSG00000248383	5	PCDHAC1	1	140926369	141012344
199	ENSG00000253276	7	CCDC71L	-1	106656765	106660996
200	ENSG00000255537	11	AP000708.1	1	125495214	125499528

Table S2: Top 200 significant genes from the one-sided Wilcoxon test that compares brain-vs-brain scores and brain-vs-heart scores.

9.2 Tissue-specific genes receive lower p-values in Wilcoxon test

From the boxplots in Figure S5, we can see that brain-specific genes and heart-specific genes receive lower p-values from the one-sided Wilcoxon test that compares brain-vs-brain alignment scores and brain-vs-heart alignment scores. When comparing heart-vs-heart scores and heart-vs-brain scores, heart-specific genes have much lower p-values. These results indicates that EpiAlign can correctly capture cell-type-characteristic chromatin state patterns.

	Gene.stable.ID	Gene.name	Chromosome	Gene.description
1	ENSG00000012660	ELOVL5	6	ELOVL fatty acid elongase 5
2	ENSG00000111832	RWDD1	6	RWD domain containing 1
3	ENSG00000112232	KHDRBS2	6	KH RNA binding domain containing, signal transduction associated 2
4	ENSG00000135968	GCC2	2	GRIP and coiled-coil domain containing 2
5	ENSG00000136485	DCAF7	17	DDB1 and CUL4 associated factor 7
6	ENSG00000138035	PNPT1	2	polyribonucleotide nucleotidyltransferase 1
7	ENSG00000138398	PPIG	2	peptidylprolyl isomerase G
8	ENSG00000139053	PDE6H	12	phosphodiesterase 6H
9	ENSG00000168958	MFF	2	mitochondrial fission factor
10	ENSG00000170293	CMTM8	3	CKLF like MARVEL transmembrane domain containing 2
11	ENSG00000173572	NLRP13	19	NLR family pyrin domain containing 13
12	ENSG00000197360	ZNF98	19	zinc finger protein 98

Table S1: Genes not on chromosome X among the top 200 genes with the smallest p-values from comparing male-vs-male scores and male-vs-female scores.

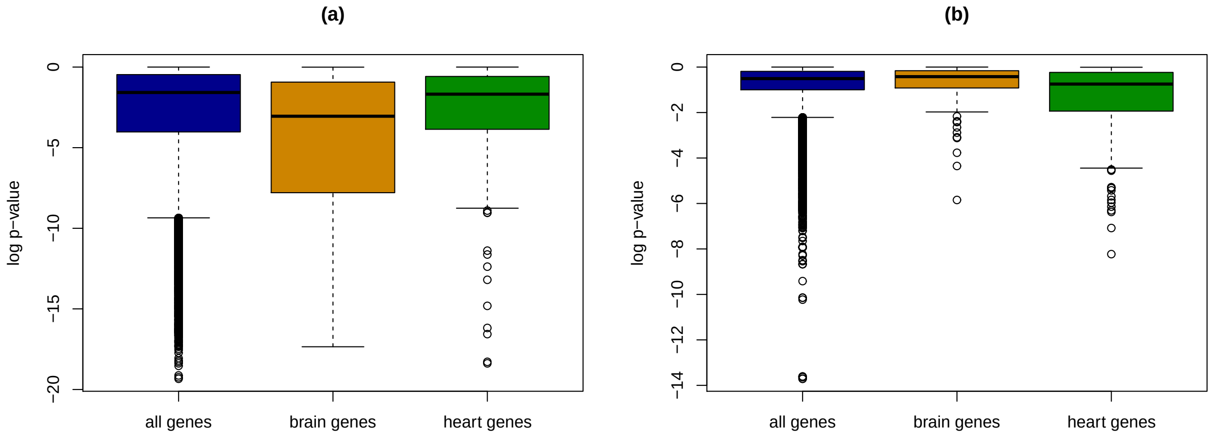

Figure S5: Boxplots of p-values of all genes, brain-specific genes and heart-specific genes from Wilcoxon tests which compares (a) brain-vs-brain scores with brain-vs-heart scores or (b) heart-vs-heart scores with brain-vs-heart scores.

10 Motif analysis

The top 200 regions with the highest horizontal alignment scores and the cluster index to which each region belongs are listed in Table S3. We use the motif-discovery tool MEME and find that all the four clusters are characterized by certain motifs, the top motifs reported by MEME are:
Cluster 1: “ihihihihihihihihihio”; “edegbabg”; “aehihedhdeo”.
Cluster 2: “gegegogegegogogegege”; “gegegegegegegegegege”; gegegegegegegegegege”;
Cluster 3: “goglkjklnog”; “dededededededededede”; “gegegegedegededegege”
Cluster 4: “oaogegegegbgege”; “mlklmlmlklklklk”; “nogogogo”.

	sample	chromosome	start.position	end.position	cluster
1	E003	chr22	22800001	23300000	1
2	E003	chr21	10700001	11200000	1
3	E003	chrX	61500001	62000000	1
4	E003	chr17	46400001	46900000	1
5	E003	chr20	59400001	59900000	1
6	E003	chr12	54100001	54600000	1
7	E003	chr12	37900001	38400000	1
8	E003	chr7	26900001	27400000	1
9	E003	chr2	176700001	177200000	1
10	E003	chr3	61500001	62000000	1
11	E003	chr6	157100001	157600000	1
12	E003	chr8	43400001	43900000	1
13	E003	chr16	33800001	34300000	1
14	E003	chr15	99100001	99600000	1
15	E003	chr11	2300001	2800000	1
16	E003	chrY	9900001	10400000	1
17	E003	chr2	92200001	92700000	1
18	E003	chr8	128600001	129100000	1
19	E003	chr7	61700001	62200000	2
20	E003	chr7	101300001	101800000	2
21	E003	chr5	89900001	90400000	2
22	E003	chr22	22300001	22800000	2
23	E003	chr4	78900001	79400000	2
24	E003	chr18	60100001	60600000	2
25	E003	chr10	34600001	35100000	2
26	E003	chr16	49400001	49900000	2
27	E003	chr6	15200001	15700000	2
28	E003	chr22	33900001	34400000	2
29	E003	chr5	54300001	54800000	2
30	E003	chr10	42200001	42700000	2
31	E003	chr14	89700001	90200000	2
32	E003	chr2	153200001	153700000	2
33	E003	chr2	121300001	121800000	2
34	E003	chr1	164300001	164800000	2
35	E003	chr19	37500001	38000000	2
36	E003	chr1	8400001	8900000	3
37	E003	chr4	190400001	190900000	2
38	E003	chr1	64200001	64700000	2
39	E003	chr5	106600001	107100000	2
40	E003	chr10	12000001	12500000	2
41	E003	chr5	13900001	14400000	2
42	E003	chr7	154900001	155400000	2
43	E003	chr11	31700001	32200000	2
44	E003	chr4	183900001	184400000	2
45	E003	chr12	132600001	133100000	2
46	E003	chr2	55200001	55700000	2

47	E003	chr8	131200001	131700000	2
48	E003	chr8	142200001	142700000	2
49	E003	chr17	59700001	60200000	2
50	E003	chr12	34400001	34900000	2
51	E003	chr11	12500001	13000000	2
52	E003	chr15	57300001	57800000	2
53	E003	chr20	49200001	49700000	2
54	E003	chr10	114400001	114900000	2
55	E003	chr5	87600001	88100000	2
56	E003	chr17	28800001	29300000	2
57	E003	chr22	29000001	29500000	2
58	E003	chr7	105300001	105800000	2
59	E003	chr22	17600001	18100000	2
60	E003	chr19	12800001	13300000	2
61	E003	chr12	32100001	32600000	2
62	E003	chr2	236200001	236700000	2
63	E003	chr3	185400001	185900000	2
64	E003	chr12	130400001	130900000	2
65	E003	chr15	26800001	27300000	2
66	E003	chr16	46000001	46500000	2
67	E003	chr11	107800001	108300000	2
68	E003	chr6	148900001	149400000	2
69	E003	chr4	93300001	93800000	2
70	E003	chr9	128200001	128700000	2
71	E003	chr15	50600001	51100000	2
72	E003	chr17	3900001	4400000	2
73	E003	chr1	235200001	235700000	2
74	E003	chr9	140300001	140800000	2
75	E003	chr19	1400001	1900000	2
76	E003	chr10	88400001	88900000	2
77	E003	chr15	28200001	28700000	2
78	E003	chr3	31600001	32100000	2
79	E003	chr2	188900001	189400000	2
80	E003	chr22	31800001	32300000	2
81	E003	chr15	44600001	45100000	2
82	E003	chr4	85400001	85900000	2
83	E003	chr13	98700001	99200000	2
84	E003	chr13	28300001	28800000	2
85	E003	chr9	33100001	33600000	2
86	E003	chr15	63800001	64300000	2
87	E003	chr1	39500001	40000000	2
88	E003	chr10	80500001	81000000	2
89	E003	chr3	121100001	121600000	2
90	E003	chr13	41000001	41500000	2
91	E003	chr16	89400001	89900000	2
92	E003	chr2	32400001	32900000	2
93	E003	chr1	219900001	220400000	2
94	E003	chr2	119400001	119900000	2

95	E003	chr1	10500001	11000000	2
96	E003	chr1	200200001	200700000	2
97	E003	chr12	112300001	112800000	2
98	E003	chr14	99600001	100100000	2
99	E003	chr8	30000001	30500000	2
100	E003	chr10	74800001	75300000	2
101	E003	chr13	113300001	113800000	2
102	E003	chr10	102800001	103300000	2
103	E003	chr18	52900001	53400000	2
104	E003	chr15	59300001	59800000	2
105	E003	chr16	1400001	1900000	2
106	E003	chr16	81200001	81700000	2
107	E003	chr8	102500001	103000000	2
108	E003	chr6	56300001	56800000	2
109	E003	chr13	100200001	100700000	2
110	E003	chr2	109000001	109500000	2
111	E003	chr10	126400001	126900000	2
112	E003	chr8	46800001	47300000	2
113	E003	chr11	126200001	126700000	2
114	E003	chr6	41100001	41600000	3
115	E003	chr2	102300001	102800000	3
116	E003	chr1	233000001	233500000	3
117	E003	chr9	16400001	16900000	3
118	E003	chr21	40200001	40700000	3
119	E003	chr9	130800001	131300000	3
120	E003	chr17	2600001	3100000	3
121	E003	chr10	96800001	97300000	3
122	E003	chrX	16700001	17200000	3
123	E003	chr13	100700001	101200000	3
124	E003	chr3	65500001	66000000	3
125	E003	chr17	25400001	25900000	3
126	E003	chr15	40100001	40600000	3
127	E003	chr5	64800001	65300000	3
128	E003	chr9	94800001	95300000	3
129	E003	chr9	124000001	124500000	3
130	E003	chr17	55300001	55800000	3
131	E003	chr22	43300001	43800000	3
132	E003	chr1	155500001	156000000	2
133	E003	chr9	125400001	125900000	2
134	E003	chr4	184500001	185000000	2
135	E003	chr20	35600001	36100000	3
136	E003	chr1	17700001	18200000	3
137	E003	chr9	70100001	70600000	3
138	E003	chr8	97400001	97900000	3
139	E003	chr22	40400001	40900000	3
140	E003	chr8	141600001	142100000	3
141	E003	chr12	11600001	12100000	3
142	E003	chr9	37600001	38100000	3

143	E003	chr8	102000001	102500000	3
144	E003	chr9	23700001	24200000	3
145	E003	chr22	45200001	45700000	3
146	E003	chr3	171700001	172200000	3
147	E003	chr15	90900001	91400000	3
148	E003	chr1	161800001	162300000	3
149	E003	chr15	42300001	42800000	3
150	E003	chr11	63600001	64100000	3
151	E003	chr1	21500001	22000000	3
152	E003	chr1	179800001	180300000	3
153	E003	chr5	46000001	46500000	3
154	E003	chr10	79200001	79700000	3
155	E003	chr18	55600001	56100000	3
156	E003	chr4	68300001	68800000	3
157	E003	chr7	121900001	122400000	3
158	E003	chr17	30200001	30700000	3
159	E003	chr11	61300001	61800000	3
160	E003	chr5	70500001	71000000	3
161	E003	chr2	202700001	203200000	3
162	E003	chr6	136500001	137000000	3
163	E003	chr1	23700001	24200000	3
164	E003	chr2	106100001	106600000	3
165	E003	chr4	48900001	49400000	3
166	E003	chr2	183700001	184200000	3
167	E003	chr6	21600001	22100000	2
168	E003	chr2	43400001	43900000	3
169	E003	chr16	72800001	73300000	3
170	E003	chr19	9200001	9700000	3
171	E003	chr1	32100001	32600000	3
172	E003	chr17	15600001	16100000	3
173	E003	chr17	27000001	27500000	3
174	E003	chr6	168200001	168700000	2
175	E003	chr6	37200001	37700000	2
176	E003	chr11	48000001	48500000	4
177	E003	chr10	7900001	8400000	4
178	E003	chr1	12300001	12800000	4
179	E003	chr9	111600001	112100000	4
180	E003	chr12	124900001	125400000	4
181	E003	chr7	55500001	56000000	4
182	E003	chr16	69000001	69500000	4
183	E003	chr15	43000001	43500000	4
184	E003	chr3	47400001	47900000	4
185	E003	chr10	70500001	71000000	4
186	E003	chr7	2400001	2900000	4
187	E003	chr4	7800001	8300000	4
188	E003	chr11	50300001	50800000	4
189	E003	chr6	166800001	167300000	4
190	E003	chr18	74600001	75100000	4

191	E003	chr5	139800001	140300000	4
192	E003	chr14	77300001	77800000	4
193	E003	chr11	121100001	121600000	4
194	E003	chr15	35000001	35500000	4
195	E003	chr6	29400001	29900000	4
196	E003	chr5	31400001	31900000	4
197	E003	chr7	23000001	23500000	4
198	E003	chr1	47800001	48300000	4
199	E003	chr10	13700001	14200000	4
200	E003	chr12	2900001	3400000	4

Table S3: The top 200 regions with the highest horizontal alignment scores are well partitioned into four clusters by average-linkage hierarchical clustering. The last column of the table is the cluster index to which each region belongs.

11 GO analysis in cross-species application of EpiAlign

We obtain mouse-human homologous gene pairs from Ensembl BioMart and sort the mouse genes with lengths 200-400 kb by gene lengths and divide the homologous gene pairs into 12 groups each with 50 pairs. We look at the molecule function GO terms of the homolog pairs that have the highest alignment scores in each group. The result (Table S4) shows that homolougous genes with high alignment scores are also very similar in molecule function. The result also indicates that EpiAlign can identify homologous genes whose epigenetic patterns are more conserved in evolution, shedding new insights into translating scientific discoveries in mice into humans.

12 Biological discovery based on top 8-mers

[2] We thank the referee for this comment, we have made the following discovery and have revised our manuscript on page 10 to discuss the potential improvement of EpiAlign: We have the following interesting findings when looking at the most common 8-mers in chromatin states identified by ChromHMM: we count the occurrence number of all the 8-mer strings from the epigenome of ESC sample E003. We look at the most frequently occurred 8-mer strings containing active TSS state (represented by 'a'). The most frequent 8-mer is 'aedededе', where 'e' represents weak transcript and 'd' represents strong transcript. This 8-mer can be interpreted as active gene region. However, there is also another 8-mer 'oioaoioi', which frequently occurs but is hard to interpret. Here, 'o' represents quiescent/low state and 'i' represents heterochromatin. We select all ESC, heart and brain samples and inspected the overlap between known TSS and these two 8-mers. The results show that in all these samples, a high proportion (71% on average) of the 8-mer 'aedededе' discovered have an overlap with known TSS while only a small proportion (28% on average) of the 8-mer 'oioaoioi' have an overlap with known TSS. This result indicates that some of the state 'a' in 'oioaoioi' may be noise.

References

[1] Yang Yang, Yu-Cheng T Yang, Jiapei Yuan, Zhi John Lu, and Jingyi Jessica Li. Large-scale mapping of mammalian transcriptomes identifies conserved genes associated with different cell

Homologous pair		GO: molecule function	
Human	Mouse	Human	Mouse
AK5	AK5	nucleotide binding	nucleotide binding
GABRB2	GABRB2	transmembrane signaling receptor activity	transmembrane signaling receptor activity
CDH2	CDH2	calcium ion binding	calcium ion binding
GAB2	GAB2	transmembrane receptor protein tyrosine kinase adaptor activity	transmembrane receptor protein tyrosine kinase adaptor activity
NEB	NEB	actin binding	actin binding
CAMK4	CAMK4	nucleotide binding	nucleotide binding
PAPPA2	PAPPA2	metalloendopeptidase activity	metalloendopeptidase activity
CNTN1	CNTN1	protein binding	protein binding
DNAH7	DNAH7b	microtubule motor activity	microtubule motor activity
TMEM132C	TMEM132C	not available	not available
SPATA16	SPATA16	not available	not available
ADCY2	ADCY2	nucleotide binding	nucleotide binding
Homologous pair		GO: biological process	
Human	Mouse	Human	Mouse
AK5	AK5	nucleobase-containing compound metabolic process	nucleobase-containing compound metabolic process
GABRB2	GABRB2	ion transport	ion transport
CDH2	CDH2	cell morphogenesis	cell adhesion
GAB2	GAB2	transmembrane receptor protein tyrosine kinase signaling pathway	transmembrane receptor protein tyrosine kinase signaling pathway
NEB	NEB	muscle organ development	regulation of actin filament length
CAMK4	CAMK4	adaptive immune response	protein phosphorylation
PAPPA2	PAPPA2	regulation of cell growth	proteolysis
CNTN1	CNTN1	cell adhesion	cell adhesion
DNAH7	DNAH7b	microtubule-based movement	microtubule-based movement
TMEM132C	TMEM132C	not available	not available
SPATA16	SPATA16	not available	not available
ADCY2	ADCY2	renal water homeostasis c	AMP biosynthetic process

Table S4: The top 1 GO terms (both molecule function and biological process) of the homolog pairs that have the highest alignment scores in each group.

states. *Nucleic acids research*, 45(4):1657–1672, 2017.

[2] W James Kent, Charles W Sugnet, Terrence S Furey, Krishna M Roskin, Tom H Pringle, Alan M Zahler, and David Haussler. The human genome browser at ucsc. *Genome research*, 12(6):996–1006, 2002.
